# Interaction of Alcohol Use and Specific Types of Smoking on the Development of Oral Cancer

**DOI:** 10.5812/ijhrba.12120

**Published:** 2014-03-11

**Authors:** Abdoul Hossain Madani, Madhurima Dikshit, Debanshu Bhaduri, Teamur Aghamolaei, Seyed Hamid Moosavy, Ali Azarpaykan

**Affiliations:** 1 Department of Public Health, Research Center for Social Determinants in Health Promotion, Hormozgan University of Medical Sciences, Bandarabbas, IR Iran; 2 Biochemistry Division, Department of Chemistry, Pune University, Pune, India; 3 Morbai Naraindas Budhrani Cancer Institute, Department of Oncology, Inlaks & Budhrani Hospital, Pune, India; 4 Research Center for Infectious Diseases, Department of Internal Medicine, Hormozgan University of Medical Sciences, Bandarabbas, IR Iran

**Keywords:** Alcohols, Smoking, Mouth Neoplasms

## Abstract

**Background:**

Oral cancer is one of the most common life threatening diseases all over the world, in particular in Asian countries, and tobacco and alcohol are considered to be the most potent risk factors for oral cancer.

**Objectives:**

The aim of this study was to examine the combined effect of smoking types and alcohol consumption on the development of oral cancer.

**Patients and Methods:**

A case-control study of 350 cases and 350 controls over a period of 19 months in the time period between February 2005 and September 2006 was carried out in Pune, India. The self-reported information about their consumption of alcohol and smoking behaviors was collected by structured questionnaires. The data was analyzed by SPSS software package. Risk analysis was performed using conditional logistic regression, which provides results in the form of crude odd ratios.

**Results:**

The smoking as well as alcohol drinking rates in the subjects of the case group were signiﬁcantly higher than the controls. Of smoking types, bidi (a hand rolled cigarette) (OR = 4.1, 95% CI = 2.4-6.9), and among alcohol types, hard liquor (OR = 2.6, 95% CI = 1. 4-6.4), country liquor (OR = 2.5, 95% CI = 1.3-3.6) and beer (OR = 2.2, 95% CI = 1.2-5.0), showed a strong association with oral cancer. A significant interaction effect was found between alcohol consumption and bidi smoking (OR = 19.6, 95% CI = 4.6-83.5) followed by alcohol and non-filtered cigarette (OR = 4.2, 95% CI = 1.8-12.0) as well as filtered-cigarette (OR = 2.3, 95% CI = 1.1-5.0).

**Conclusions:**

We conclude that oral cancer is etiologically related to the interaction between smoking and drinking.

## 1. Background

Oral cancer is the eighth most common cancers in the world accounting for approximately 4% of new cancer cases and 2% of all cancer deaths worldwide (1, 2). Alcohol, smoking and smokeless tobacco increase the risk of oral and pharyngeal cancers (3, 4). In India, where chewing and smoking of tobacco is practiced, there is a strikingly high incidence of oral cancer- which account for as many as 50% of all cancers (5).

Smoking of all cigarette types such as bidi (a crude form of cigarette with about 0.2 gram of tobacco wrapped in a specific tree leaf), cigar and pipe has been reported to be a risk factor for oral cancer (6).

Many epidemiological studies conducted over the last three decades in America, Europe, and Asia have provided strong evidence of association between alcohol and tobacco use and increased risk of oral and pharyngeal tumors (7-11).

Despite this information, there remain several important issues to be clarified, including the determination of combined effects of alcohol and smoking on oral cancer. Moreover, what is the estimation of the magnitude of the effect? Thus in this study we investigated how smoking types and alcohol consumption will interactively affect the development of oral cancer.

## 2. Objectives

This study was conducted to investigate the combined effect of alcohol drinking and smoking on oral cancer; in this study the basic question addressed was: do these two exposures pose their risks in some interactive mode?

## 3. Materials and Methods

A case-control study was conducted at Pune, Morbai Naraindas Budhrani Cancer Institute within 19 months from February, 2005 to September, 2006. A total of 700 age and gender matched subjects including 350 cases and 350 controls were selected using simple random sampling. Cases were the newly diagnosed patients of oral cancer aged above 18 years.

The control subjects were selected from the relatives, friends and caretakers of case subjects, who accompanied the patients at the hospital and were healthy and did not reportedly have cancer. A written consent form was obtained from each participant. A trained interviewer interviewed the case and control participants when the situation was appropriate.

We used structured questionnaires to obtain complete information on demographical characteristics such as age, gender, education, income, place of residency, occupational history, religion, marital status, tobacco-related behavior, alcohol consumption and dietary habits. The individuals who used smokeless tobacco (such as paan, supari and gutka) were not included for un-adjusted odds ratio analysis in this study. The significance of differences between the proportions of qualitative characteristics were tested using Chi-square test. The quantitative risk assessment was done by calculating the odds ratios (OR) with 95% confidence intervals. The entire data was analyzed using the Statistical Package for Social Sciences (SPSS) version 14.

## 4. Results

The self-reported age at the time of data collection (interview) ranged from 18 to 80 years among cases and controls. The average age of case and control subjects was approximately similar (52.4 years and 51.8 years, P = 0.551 using students’ ‘t’ test) and majority of the cases and controls were older than 40 years (77.7% vs. 78.6% P = 0.780). There were 251 (71.7%) male and 99 (28.3%) female cases, the sex ratio being 2.5:1. There were 254 (72.6) male and 96 (27.4) female controls, the sex ratio being 2.6:1. Thus, the distribution of sex was also similar between the case and control groups (P = 0.800 by Chi-Square test). Seventy-three percent of the cases were urban and semi-urban residents and remaining (27%) were rural residents. Approximately similar distribution was observed in the control group. These findings indicate that the control and case groups were matched in terms of age, gender and their residential status (Table 1). 

**Table 1.** The Demographic Characteristics of the 700 Studied Cases and Controls ^a^

	Cases	Controls	P value
**Gender**			
Male	251 (71.1)	254 (72.6)	0.8
Female	99 (28.3)	96 (26.4)	
**Age **			
< 40	78 (22.3)	75 (21.4)	0.78
41-50	85 (24.3)	76 (21.7)	
51-60	94 (26.9)	104 (29.7)	
61 ^+^	93 (26.6)	95 (27.1)	
**Location**			
Rural	94 (26.9)	87 (24.9)	0.4
Urban & semi urban	256 (73.1)	263 (75.1)	

^a^ Data are expressed as No. (%), (n = 350).

Over-all, smoking as well as drinking rates were significantly different between cases and controls (35.7% vs. 17.4% P < 0.001, 30.3% vs. 13.7%, P < 0.001, respectively).

Smoking was further categorized into three subtypes, i.e. filtered cigarette, non-filtered cigarette and bidi (hand-rolled locally available cigarette), the prevalence of which was significantly higher in case group compared to control group (12.6% vs. 9.4%, 4.3% vs. 1.7%, and 20.0% vs. 5.7%, P < 0.001 for all respectively). In terms of consumption of alcoholic beverages, subjects in the case group consumed significantly more country liquor, hard liquor and beer (P < 0.05 for all) (Table 2). 

**Table 2.** Distribution of Subjects by Selected Risky Behavior ^a^,^b^

	Cases	Controls	P value
**Smoking (overall)**	125 (35.7)	61 (17.4)	0.001
**Filtered cigarette**	44 (12.6)	33 (9.4)	0.149
**Non filtered cigarette**	15 (4.3)	6 (1.7)	0.046
**Bidi (hand rolled cigarette)**	70 (20)	20 (5.7)	0.001
**Alcohol (overall)**	106 (30.3)	48 (13.7)	0.001
**Bear**	28 (8)	14 (4)	0.026
**Wine**	4 (1.1)	6 (1.7)	0.524
**Hard liquor**	29 (8.3)	10 (2.9)	0.002
**Country liquor**	55 (15.7)	25 (7.1)	0.001

^a^ Values are No. (%), P-by Chi-square test.

^b^ Abbreviation: NS, not significant.

The odds ratio derived by univariate analysis suggest that, overall, smoking and alcohol drinking were significant risk factors for oral cancer. Regarding the types of smoking, risk of oral cancer was found to be maximum among the bidi smokers (OR = 4.1, 95% CI, 2.4-6.9) followed by non-filtered cigarette smokers. Alcolohol consumption types were further categorized into beer, hard liquor, country liquor and wine. Hard-liquor drinkers had significantly increased risk of oral cancer compared to those who did not take hard liquor regularly (OR = 2.6, CI = 1.4-6.4). The risk was also significant among those who drank the country liquor and beer regularly, while it is interesting to note that the filtered cigarette and drinking of wine alone were not significantly associated with oral cancer (Table 3). 

**Table 3.** Odds Ratios (Unadjusted OR) for Types of Active Smoking and Alcohol ^a^

	Cases, No.	Controls, No.	OR	95% CI of OR	P value
**Smoking overall**	125	61	2.6	1.8-3.7	0.001
**Filtered cigarette **	45	33	1.4	0.8-2.3	0.149
**Non-filtered cigarette **	15	6	2.5	1-6.7	0.046
**Bidi**	70	20	4.1	2.4-6.9	0.001
**Alcohol overall**	106	45	3	1.9-4.3	0.001
**Beer**	29	12	2.2	1.2-5	0.026
**Hard liquor**	29	10	2.6	1.2-5.5	0.002
**Country liquor**	55	25	2.5	1.3-3.6	0.001
**Wine**	12	7	1.7	0.6-4.3	0.524

^a^ Un-adjusted OR, P value by Ch-Square test, 95% CI of OR < 1 = Not significant.

Among consumers of both alcohol and tobacco, the increased risk of oral cancer showed a more multiplicative rather than additive mode. Combined bidi smokers and alcohol drinkers had significantly higher risk of oral cancer (OR = 19.6 (CI = 4.6-83.5)) followed by non-filtered and filtered cigarette smokers and alcohol (OR = 4.2 (CI = 1.8-12.0)) and OR = 2.3 (CI = 1.1-5.0), respectively). A remarkable finding was that combination of bidi smoking with alcohol increases the risk of oral cancer approximately by five folds compared to the bidi smoking alone (19.6 vs. 4.1); while, filtered cigarette smoking was a significant risk for the development of oral cancer only when used in combination with alcohol (OR = 2.3 (1.1-5.0)) (Tables 4). 

**Table 4.** Odds Ratios (Unadjusted OR) of Oral Cancer for the Combined Use of Smoking and Alcohol ^a^

	Cases, No.	Controls, No.	OR	95% CI of OR	P value
**Overall smoking and alcohol**	102	32	23.7	12.6-44.6	0.001
**Filtered cigarette and alcohol**	20	11	2.3	1.1-5	0.026
**Non-filtered cigarette and alcohol**	14	4	4.2	1.8-12	0.003
**Bidi and alcohol**	28	2	19.6	4.6-83.5	0.001

^a^ Un-adjusted OR, P-by Chi-Square test, 95% CI of OR < 1 = Not significant.

## 5. Discussion

In general, smoking was more common among the case subjects compared with controls and of smoking types, bidi was more frequent which is in line with several epidemiological studies suggesting that bidi smoking increases the risk of oral cancer (4, 12-14). We found an increased risk of oral cancer with bidi smoking compared with other type of smoking. Similar results have been reported before in India, which may be due to the higher content of nicotine in bidi (14-16).

In the present study alcohol use was a significant risk factor for oral cancer. However, risk for hard liquor was more than the others. Similar findings are reported from a case-control study of three areas in Southern India (17). Alcohol is thought to play a direct causal role in the development of oral cancer by affecting oncogenes that play a role in the initiation and progression stages of oral cancer. This is carried out by impairing DNA damage repair mechanisms and by over expression of certain oncogenes, which trigger cancer progression. The dehydrating effect of alcohol on cell walls enhances the ability of tobacco carcinogens to permeate mouth tissues; also the nutritional deficiencies associated with heavy drinking can lower the body’s natural ability to use antioxidants to prevent the formation of cancer. Specific alcoholic beverages have been shown to contain certain chemical impurities like N-nitrosodiethylamine in beers and polycyclic aromatic hydrocarbons in some brands of whiskey which have proved to be carcinogenic (18, 19).

When we analyzed the effect of smoking types and concurrent alcohol consumption, the risk of oral cancer was significantly increased compared to using either alcohol or tobacco alone, thus these two substances have significant interactive role in causing oral cancer.

Of the smoking types, combination of bidi and the alcohol was the strongest risk factor for oral cancer which is in line with other studies (12, 20). However, the evidence for the interaction of bidi and alcohol in causing oral cancer is inconsistent (17, 18). This inconsistency might be attributed to the fact that the previous studies did not control for alcohol consumption, which is an independent risk factor for oral cancer and therefore could be a strong confounding factor.

Combining smoking and excessive alcohol intake, increases the risk of developing oral cancer, and we estimate that in our study , 30% of all cases of oral cancer were developed due to the combined effect of smoking and alcohol use. This could be because, alcohol dissolves certain compounds in the tobacco smoke which are linked to cancer and/or alcohol increases the permeability of the epithelium inside the mouth.
